# Musculoskeletal disorders among nursing staff: a comparison of five hospitals in Uganda

**DOI:** 10.11604/pamj.2014.17.81.3213

**Published:** 2014-01-31

**Authors:** Ian Guyton Munabi, William Buwembo, David Lagoro Kitara, Joseph Ochieng, Rose Chalo Nabirye, Erisa Sabakaki Mwaka

**Affiliations:** 1 Anatomy Department, School of Biomedical Sciences, Makerere University College of Health Sciences, Uganda; 2 Surgery Department, Gulu University, Uganda; 3 Department of Nursing, School of Medicine, Makerere University College of Health Sciences, Uganda

**Keywords:** Musculoskeletal disorders, nurses, public and private hospitals, Uganda

## Abstract

**Introduction:**

Low and middle income countries have severe nursing staff shortages which is associated with risk of poor quality of patient care and increased patient exposure to adverse events. This is accompanied with increased risk of musculoskeletal disorders to the nursing staff. This paper sets out to identify and compare factors associated with musculoskeletal disorders among nursing staff in 5 different hospitals in Uganda.

**Methods:**

This was a cross sectional study on nurses from 5 different hospitals in Uganda. The study used a 12 month recall of reported Musculoskeletal disorders (MSD) among nurses. Ethical approval was obtained. Logistic regression analysis and ANOVA were used. The level of significance was set at 0.05 for all statistical tests.

**Results:**

There were 755 respondents of whom 433 (58.4%) were nurses. The prevalence of MSD at anybody site was 80.8%. There were significant differences in reported MSD among nursing staff across different hospital settings which were worse in the public hospitals as compared to the private and private not for profit hospitals (p <0.001). Age (adjusted OR 1.03, 95% CI 1.01-1.06), self reported poor general health status (adj OR 4.5, 95% CI 2.8-7.24) and stress as suggested by waking up tired in the morning (adj OR 3.4, 95% CI 2.17-5.32) were significant associated factors for MSD in this population.

**Conclusiom:**

Reported MSD among nursing staff across 5 different hospitals is worse in public as compared to private hospitals. Age, self reported poor general health status and stress were important factors for MSD in this population.

## Introduction

Africa bears 25% of the world ‘s disease burden yet it has only 3% of the world ‘s health workers and 1% of the world ‘s economic resources to meet that challenge [1]. The Millennium Development Goals identified 57 countries with critical shortages of health workers-36 of these countries are in Africa [1]. In Uganda it is reported that the shortage of nursing professionals currently stands at a dismal six (6) nurses per 100,000 population compared to 773 nurses per 100,000 population in the USA[2]. Shortage of nurses leads to increased workload which results in occupational stress among the nurses. Occupational stress has been identified as one of the leading causes of work related injuries, including musculoskeletal disorders (MSD)[3] In most Ugandan hospitals (both private and public) nurses work in 8 hour shifts. In response to shortages in nursing staff, Mulago National Referral and Teaching Hospital of Uganda recently announced a proposal to change from the 8 hour working shifts to 12 hour working shifts for its nursing care staff [4]. Whereas the proposal was initially rejected by the local nursing staff in Uganda, in more developed countries, the 12 hour shifts were well received initially compared to the shorter 8 or 10 hour shifts [5, 6]. In these developed countries, the 12 hour shift was initially associated with more time for family and other non health facility issues of importance to the nurses [6–8]. It is reported that they could not find this time when working the shorter and more frequent shifts in their 35-40 hour work weeks [6–8]. Most of the recent publications show that the quality of patient care in developed countries with the 12 hour shift is going down [9]. The evidence for this is the observed higher frequency of documentation errors [10] and the increased risk of patient exposure to adverse events [11]. For the nurses the longer 12 hour shifts have been associated with the development of various forms of stress [12], fatigue [10, 13], and altered body physiology [14]. Beyond the fatigue, research now demonstrates that the nursing staffs on these longer shifts are more prone to developing occupational injuries including MSDs [15]. The working environment and options given to nurses to choose how they work play an important role in mitigating the effects of the long working shift hours [16, 17]. In low resource countries, nurses have little or no job control, which is another source of stress. With severe nursing staff shortage in Uganda, there is a need to document the effect of the nurses work schedule on nurses in order to provide a baseline for future comparisons and eventual planning [13, 15, 18]. To our knowledge such documentation is still lacking. In addition, the variety in management of health facilities creates variations in the working environments between the different categories of hospitals (private for profit, private not for profit and public), in Uganda. This paper sets out to compare the effect of different hospital settings on reported musculoskeletal disorders among nursing staff in Uganda.

## Methods

This was a cross sectional study conducted among nursing staff from 5 hospitals in Uganda. These hospitals included; one private for profit hospital located in the capital city (A), 3 regional referral public hospitals (B, C, D) located in central, northern and eastern Uganda respectively, and one private not for profit hospital (E) located in the capital city. All nursing staff in the study hospitals were eligible for inclusion in the study. For this paper, nursing staff encompassed nurses, midwives and nursing assistants/aides. Midwives and nursing assistants were included in the study because they perform nursing care duties in our hospitals. Because of the lack of nursing staff especially in Ugandan public hospitals, midwives and nurses are double trained and together with nursing assistants perform general nursing and midwifery duties, and are hence equally exposed to manual patient handling activities.

### Questionnaire design

Data was gathered by means of an anonymous, self-reporting questionnaire which was adapted from the standardized Dutch Musculoskeletal (DMQ) and Nordic Musculoskeletal questionnaires (NMQ) [19, 20]. These questionnaires have been used extensively in research studies on MSD in the general population and among different occupational groups [21–27]. The use of these questionnaires for assessing MSD has also been validated in other publications and shown to be acceptable [20, 28–32]. The DMQ is a validated tool for the analysis of musculoskeletal workload and associated potential hazardous working conditions as well as musculoskeletal symptoms in worker populations. The DMQ enables a global assessment of musculoskeletal workload and other potentially hazardous working conditions by seven homogeneous indices (forces, dynamic loads, static loads, repetitive loads, climatic factors, vibration and ergonomic environmental factors) and four separate factors (sitting, standing, walking, uncomfortable postures). The questionnaire was pre-tested, simplified and adjusted to our local situation for easy understanding. It comprised of a simple tick-box format, with the first section covering demographic (individual) items such as age, tobacco smoking, alcohol consumption, marital status, and number of children. The second section focused on workplace factors such as work hours, shift work, and career duration as a nursing professional. Questions addressing the general health status and psychosocial factors constituted the third component, and were adapted from the DMQ [19]. Further items included their daily physical tasks, posture, and perceived intensity of work.

MSD were classified according to the criteria of Kuorinka et al [20], who defined them as an ache, pain,discomfort or numbness in the defined area over a set period of time. MSD questions included an anatomical diagram with specifically shaded areas, which focused on the occurrence of symptoms at certain body sites over the previous 12-month period and whether sick leave had been taken or medical advice sought [20]. A 12-month recall period was used for MSD, as this has been shown to be an appropriate time-scale in other studies [21] Permission of the administrators of the selected research centers was obtained before the commencement of the study. Trained research assistants together with the nursing directors in the respective hospitals assisted in the consecutive distribution of the questionnaire. They, together with the research team guided the respondents during the completion of the questionnaires.

Data was previously entered into Epidata version 3.2 (Epidata association, Denmark) and exported to STATA 12 (StataCorp LP, Texas, USA) where after checking for duplicate entries a preliminary analysis of each variable was made to identify additional range and omission errors. The aim of the analysis was to identify the factors predicting MSD at the different hospitals. This was guided by an analytical model using the age of respondent and hospital location as the core determinants of reported MSD. Uni-factorial and multi-factorial logistic regression were done to identify other significant associations of reported MSD in the study population. ANOVA was used to assess the differences in means among the 5 hospitals. The results of the analysis were then summarized using frequencies, means, standard deviations, confidence intervals and odds ratios. The level of significance was set at 0.05 for all statistical tests. Ethical review and approval was received from the Makerere University School of Biomedical Sciences Research and Ethics Committee and the Uganda National Council of Science and Technology. Written Informed consent was obtained from all participants before enrolment into the study. The study was conducted according to the WHO guidelines required for conducting research on humans [33].

## Results

A total of 880 questionnaires were distributed to nursing staff from the 5 hospitals in Uganda, from whom 755 responded giving a response rate of 85.4%. The combined total population of nursing staff in the 5 hospital was 895. Respondents comprised of nurses (433/741, 58.4%), midwives (214/741, 28.9%), theatre staff (28/741, 3.8%) and nurse assistants (66/741, 8.9%). There were more female (647/755, 85.7%) than male (108/755, 14.3%) respondents. The average age was 35.4 years standard deviation 10.7 years (range 18-62 years), 443/744 (59.4%) were married and 645/755, (85.4%) had children. The average working hours per week was 43.7 hours SD 18.9 hours and the mean career duration of 11.9 years SD 10.5 years. About 82.6% of respondents reported inadequate tools and equipment though there was no significant difference between the different hospital categories (p = 0.16). The prevalence of MSD at anybody site among the nursing staff was 80.8%.

Baseline comparisons for; age, working environment factors, number of children, and general health for the study population across the different locations are summarized in Table 1. In this table note that the only similarity was in the number of shifts staffs had across all the hospitals. It is also important to note that hospital D seemed to have several significantly different parameters for the study variables when compared to the other hospitals. This is further demonstrated in Table 2; where this same hospital has the highest frequencies of reported MSD for the various body regions All the independent variables in Table 1 with the exception of; occupation, number of shifts, private life being affected, patient handling, having other part time jobs, sick leave and hours in the work week, significantly predicted respondents reporting MSD in the last 12 months. When these significant variables were analyzed together, the following variables became non significant: Female gender (Adj. OR 0.96 95% CI 0.49 to 1.88), number of children (Adj. OR 0.94 95% CI 0.80 to 1.09), career duration (Adj. OR 1.02 95% CI 0.95 to 1.09), feeling tense at the end of the day (Adj. OR 1.17 95% CI 0.67 to 2.05) and staff shortages in the unit (Adj. OR 1.51 95% CI 0.81 to 2.82). Table 3; summarizes the results of the analysis that was re-run retaining only the significant variables. It is demonstrated that there was a significant threefold increase in the odds of reporting MSD for respondents from hospital D with respect to hospital A. The retained variables (age, complaints about health and feeling tired on waking up in the morning) in the model remained significant with age having a 3% increase in odds for MSD per year of adult life, a fivefold increase in the odds of MSD for those reporting health complaints and threefold increase of MSD for those that reported being tired in the morning.

**Table 1:** Characteristics of study population according to their hospital

Hospital	A			B			C			D			E			ANOVA
Items	N*	Avr	SD	N*	Avr	SD	N*	Avr	SD	N*	Avr	SD	N*	Avr	SD	F (P-value)
Age (yrs)	82	29.68	5.02	164	38.04	10.37	157	36.24	12.08	150	41.04	11.84	195	30.59	7.01	33.75 (<0.0001)
Children	62	2.34	2.5	150	2.53	2.05	141	2.85	1.71	131	3.15	2.17	161	1.83	1.44	9.88 (<0.0001)
Gender**	82	0.268	0.45	166	0.13	0.33	157	0.15	0.36	152	0.14	0.35	198	0.10	0.30	3.51 (0.0075)
Occupation**	82	0.78	0.42	163	0.68	0.47	154	0.72	0.45	149	0.64	0.48	196	0.64	0.48	0.55 (0.703)
Career duration (yrs)	82	6.41	6.56	163	14.32	10.00	151	13.16	11.41	148	17.01	12.08	194	7.46	6.68	30.02 (<0.0001)
No. shifts/day	82	2.33	1.07	163	2.31	0.99	153	2.19	1.04	150	2.4	1.16	198	2.42	1.07	1.22 (0.2992)
Work week (hrs)	81	46.35	16.37	157	45.66	14.14	142	41.96	24.53	142	40.91	16.57	188	44.45	20.05	1.97 (0.0973)
Sick leave**	81	0.04	0.19	163	0.07	0.25	154	0.10	0.31	149	0.11	0.31	197	0.04	0.20	2.39 (0.0496)
Other part-time jobs**	80	0.24	0.43	163	0.12	0.33	156	0.04	0.19	148	0.11	0.31	198	0.07	0.25	7.00 (<0.0001)
Health complaints**	82	0.48	0.50	160	0.49	0.50	154	0.53	0.50	148	0.77	0.42	195	0.47	0.50	15.20 (<0.0001)
Feeing tense**	77	0.32	0.47	161	0.47	0.50	152	0.51	0.50	149	0.73	0.44	197	0.54	0.50	10.58 (0.0001)
Wake up tired**	82	0.48	0.50	161	0.51	0.50	155	0.51	0.50	150	0.72	0.45	193	0.58	0.49	5.49 (0.0002)
Staff shortage**	78	0.66	0.48	163	0.94	0.24	151	0.84	0.37	151	0.93	0.25	194	0.71	0.45	16.92 (<0.0001)
Private life affected**	79	0.58	0.49	163	0.63	0.49	151	0.55	0.50	149	0.50	0.50	196	0.66	0.48	2.59 (0.0357)
Patient handling**	83	0.93	0.26	163	0.81	0.39	152	0.80	0.40	149	0.79	0.41	197	0.91	0.29	4.36 (0.0017)

* N population sample for each location that responded to the item variability due to missing items

** Categorical items coded as 1/0; Average (Avr) therefore represents proportion of respondents to item coded as 1; In Bold print Significantly different means at < 0.05 compared to hospital A

**Table 2:** Reported MSD in the last 12 Months by Body Region According to Hospital

Hospital	A		B		C		D		E		ANOVA
Items	n**	Percent**	n*	Percent**	n*	Percent**	n*	Percent**	n*	Percent**	F (P-value)
Neck	21	25.93	40	25.81	50	33.33	66	46.81	80	41.24	8.00 (<0.0001)
Shoulder	22	27.85	39	25.32	41	28.47	63	45.99	66	34.02	4.32 (0.0019)
Upper back	18	22.78	54	34.18	57	39.31	61	43.57	66	34.02	2.69 (0.0302)
Elbows	9	11.54	25	16.13	16	11.03	32	23.53	27	14.06	2.57 (0.0367)
Wrist and Hands	13	16.46	43	27.74	44	29.73	49	36.84	57	29.53	2.56 (0.0374)
Lower back	36	45.57	89	55.97	90	61.64	111	79.29	119	61.03	7.59 (<0.0001)
Hips and Thighs	12	15.19	34	21.94	47	33.10	64	45.39	41	21.24	9.61 (<0.0001)
Knees	17	21.79	59	37.58	50	34.01	78	54.55	63	32.47	7.47 (<0.0001)
Ankles and Feet	20	25.00	61	38.61	45	30.41	63	44.68	86	44.10	3.84 (0.0043)

* n population sample for each location that responded to the item

** Percentage proportion of respondents responding positively to item at each hospital; In Bold print Significant items

**Table 3:** Factors related to reported MSD by nursing staff at the different hospitals estimated in logistic regression analysis

Variable	Odds ratio	Adj Odds ratio *	95% CI	P value
*Hospital*				0.0006 ***
Hospital A**	1.0			
Hospital B	0.87	0.60	0.3 to 1.19	
Hospital C	1.81	1.60	0.77 to 3.27	
Hospital D	6.43	2.77	1.07 to 7.14	
Hospital E	1.6	1.50	0.76 to 2.94	
Age	1.05	1.03	1.01 to 1.06	
Complaints about health	6.93	4.50	2.80 to 7.24	
Feel tired on waking up in the morning	4.98	3.40	2.17 to 5.32	

* Adjusted odds for all the other variables in the table

** Reference value for hospitals

*** Overall p-value for the model

## Discussion

We set out to compare the effect of different hospital settings on reported musculoskeletal disorders among nurses in Uganda. We found significant differences in reported MSD in the different hospitals sampled with the greatest differences being observed with Hospital D a public regional hospital (Table 1, Table 2, Table 3). It is important to note that overall, public hospitals (B,C,D) had greater odds of nursing staff reporting having had a MSD in the last 12 months than nurses from the private hospitals (A and E). A possible explanation for this observation could be the differences in age between the nursing staff in the public hospitals and those in the non public hospitals as seen in Table 1. This is further supported by other age related variables, with large difference in the two types of hospitals (Table 1) that include; career duration and number of children. In a review of work-related musculoskeletal disorders in nurses [34] older age was significantly associated with neck/shoulder MSD. Age is often correlated with job tenure, an association explained by the cumulative effects of long-term repetitive physical exposures to injury of these nurses in their work environment. In this study this pattern could be the underlying mechanism for the observations made with hospital D, which had both the highest average age among the respondents and also had the highest odds for reported MSD. In Uganda, only 63% of approved positions are filled by qualified health workers with the majority being based in urban areas[35]. This is another factor that might predispose nursing staff to MSD, the shortage of staff in the public hospitals, coupled with the very high numbers of patients leading to work overload. The 3 public hospitals in this study were at locations away from the capital city which employs up to 40% of nurses/midwives in Uganda to cover about 12% of the total population[35].

There was no significant difference in the number of shifts done by the respondents in the five hospitals (Table 1). This could suggest that the duration of work related exposure to factors leading to MSD is comparable across all the hospitals. What stands out is the observation that hospital A has a higher proportion of male nurses employed, fewer staff shortages, a larger proportion of nurses with additional part time jobs and fewer respondents who were either midwives or theatre staff. So in addition to being younger we see that hospital A may have slightly better working conditions than the other four participating hospitals. The other factor that could explain this is the fact that hospital A as a private institution would be focused on provision of quality service which goes hand in hand with the employees working environment. For economic reasons most private for profit hospitals in our country have few permanent staff and usually rely on part-timers. In Table 1; note that the proportions of nurses who reported manually handling patients was greater than 90% for hospital B, C, D, and E. This would involve hard physical work such as carrying patients off bed on to trolleys and other such related activities that place an additional strain on the body. About 82.6% of respondents reported inadequate tools and equipment though there was no significant difference between the different hospital categories. Shortage of basic equipment increases exposure to manual patient handling and awkward working postures which have been documented to be significant risk factors for MSD[3]. This calls for an assessment of the working aids available in the public hospitals to identify ways of reducing this exposure as is seen in the private setting in hospital A.

The discussions to increase the nursing staff work shifts from 8 hours to 12 hours should go hand in hand with measures to address nursing staff shortages and provision of protective work aids [4]. This is important given that in the current 8 hour scenario the nursing staff have a threefold increase of MSD for those that reported being tired in the morning, a possible pointer to occupational stress. Instead of increasing the duration of the current work shifts in public hospital the policy makers ought to think otherwise. Extended workdays may be too fatiguing if the workload is high [36, 37]. Twelve-hour shifts often have positive effects on employees’ satisfaction, but can be disadvantageous for fatigue, health and performance[38]. Beneficial health effects through the reduction of the regular workday in industry from 12 or 10 to 8 hours were first suggested at the beginning of the last century[39]. Wergeland et al reported a one third decrease in the prevalence of neck shoulder pain 1.5 years after reducing daily working hours from 8 hours to 6 hours. On a theoretical basis, a reduced duration of muscular strain can be expected to reduce the risk of musculoskeletal pain. Thus the reduced work-hours may have reduced the prevalence of neck-shoulder pain through several pathways. Also shorter workdays reduce the total energy expenditure required in physically demanding jobs, which in turn, reduces fatigue and the risk of musculoskeletal injury [40].

Also the observed fivefold increase in the odds of MSD for those reporting health complaints, suggests that most health complaints in the last 12 months could be related to MSD. These complaints increase with age at a rate of 3% increase in odds for MSD per year of adult life (Table 3). This could lead to reduced productivity as suggested in Table 1; where the nurses in hospital D with the highest odds for MSD and mean age also had the lowest mean reported hours of work per week. We postulate that an increase in the work hours per week for the current nursing staff will lead to an increase in the reported MSD and other medical errors as observed elsewhere [10, 15, 17]. In the event that the efforts to increase the work shift for nurses to 12 hours per day proceed, such a change should; put in place the relevant protective mechanisms and a support system for those nurses that do eventually develop MSD; and preferably employing many more younger nurses as was done in hospital A. This is supported by the observations from both our data and literature that show that younger nurses tend to have fewer MSD related complaints [12]. Interpretation of our study results must take into account several limitations of the study design. Errors may have occurred from biased recall of symptoms or activities and hospitals did not have a uniform number of nursing staff. The small sample of five hospitals with only two private hospitals and the results being based on, survey results of self reported data. Future studies should look at more hospitals and other sources of information like staff health records with the different human resource units of the participating hospitals. In spite of this, the number of respondents from each unit was large enough to support the drawing of indicative deductions on the effect of MSD in different hospital settings.

## Conclusion

We were able to demonstrate that there are significant differences in reported MSD among nurses across different hospital settings. This was worse in the public hospitals as compared to the private and faith based private not for profit hospitals. Age, complaints about health and stress as suggested by waking up tired in the morning were identified as significant factors associated with MSD in this population. The proposed change in policy to increase in the work hours may lead to a worsening of this situation if these issues are not addressed.
